# Monochromatic Photophase Light Alters Diurnal Profiles of Melatonin Pathway Indoles in the Rat Pineal Gland

**DOI:** 10.3390/ijms26136515

**Published:** 2025-07-06

**Authors:** Bogdan Lewczuk, Kamila Martyniuk, Natalia Szyryńska, Magdalena Prusik, Natalia Ziółkowska

**Affiliations:** Department of Histology and Embryology, Faculty of Veterinary Medicine, University of Warmia and Mazury in Olsztyn, Oczapowskiego 13, 10-719 Olsztyn, Poland; kamila.kwiecinska91@gmail.com (K.M.); natalia.skiepko@uwm.edu.pl (N.S.); mprusik@uwm.edu.pl (M.P.); natalia.trzaska@uwm.edu.pl (N.Z.)

**Keywords:** pineal gland, melatonin, N-acetylserotonin, serotonin, tryptophan, monochromatic light, light-emitting diode, biological rhythm

## Abstract

Light is a major environmental factor that regulates circadian rhythms and pineal melatonin synthesis. While the influence of nighttime light exposure on melatonin suppression has been extensively investigated, much less is known about the impact of photophase light wavelength on pineal function. The aim of the study was to determine the influence of monochromatic light during the photophase on diurnal changes in melatonin-related indoles in the rat pineal gland. Wistar rats were exposed for 7 days to 150 lx of monochromatic blue (463 ± 10 nm), green (523 ± 10 nm), or red (623 ± 10 nm) LED light, or to white fluorescent light (control), under a 12:12 light–dark cycle. Pineal glands were collected every 3 h over 24 h, and the indole content was analyzed by high-performance liquid chromatography. The results demonstrated that both the timing and course of N-acetylserotonin (NAS) and melatonin (MLT) rhythms were significantly affected by light wavelength. Blue light most effectively preserved the normal rhythmicity observed under full-spectrum white light, whereas green—and particularly red light—delayed nocturnal NAS and MLT synthesis. These changes were accompanied by concurrent alternations in rhythms of serotonin, its precursors, and metabolites. The data strongly suggest that spectral light composition during the photophase influences pineal indole metabolism via melanopsin-mediated phototransduction and possibly other retinal mechanisms. These findings may have implications for the design of artificial lighting environments in human life and animal housing.

## 1. Introduction

Light is fundamental for visual perception and image formation but also acts as a crucial regulator of numerous physiological processes in both humans and animals [1]. Nowadays, the increasing prevalence of indoor lifestyles, characterized by prolonged exposure to artificial light-emitting devices and reduced access to natural sunlight, has raised concerns regarding its impact on diurnal rhythmicity. The intensity and wavelength of light during the day, along with sufficient darkness at night, play a vital role in synchronizing biological rhythms [1,2]. Disruptions in these rhythms are linked to neuroendocrine dysregulation, metabolic disorders, elevated stress levels, and an increased risk of cancer, cardiovascular diseases, and premature death [3].

Light-emitting diodes (LEDs) are increasingly replacing traditional light sources for both indoor and outdoor illumination, as well as in the displays of electronic devices [4]. LEDs differ from tungsten and fluorescent lamps in their spectral characteristics. The white LEDs use blue light to excite phosphors to produce white light, and as a consequence, these LEDs emit much more blue light than traditional light sources. Conversely, color LEDs are monochromatic light sources; therefore, red and green LEDs are completely devoid of the blue light component.

Blue light is essential for synchronizing circadian rhythms with the environmental photoperiod [2,5,6], because light perception for regulation of daily and seasonal fluctuations of physiological processes occurs mostly via specialized photopigments such as melanopsin and pinopsin, which are most sensitive to the short-wavelength range of the visible spectrum [7,8]. Melanopsin-containing retinal ganglion cells play a pivotal role in aligning the internal clock with environmental light cues [7]. The blue light emitted by LEDs is frequently considered a factor that disturbs biological rhythms, including the sleep–wake cycle, or even causes retinal damage [9,10]. However, the proper use of blue light can enhance alertness, improve reaction times, and reduce sleepiness [11].

Melatonin (MLT), a key biochemical marker of darkness, is produced and released by the pineal gland in a diurnal rhythm tightly regulated by environmental light conditions [12]. In mammals, the photic information is transmitted from the retina via the retinohypothalamic tract to the suprachiasmatic nucleus that governs pineal activity through a multisynaptic neuronal pathway [7,13,14]. A well-established dose-dependent relationship exists between light intensity and melatonin suppression during nighttime exposure [15,16,17,18]. However, much less is known about the spectral effect of light on MLT secretion. As mentioned above, a subpopulation of retinal ganglion cells possesses the nonvisual photopigment melanopsin, and this cell population plays a crucial role in the light control of pineal activity [7,19,20,21]. Melanopsin is most sensitive to light at approximately 480 nm [7,22,23,24]; therefore, the blue light is the most effective at suppressing MLT synthesis [11,24,25,26,27,28]. Green light can also suppress MLT, though its effectiveness diminishes with prolonged exposure [28]. The effect of red light on MLT suppression is not well defined. Red light is generally considered to exert minimal or no influence on the daily rhythm of melatonin secretion and is frequently utilized as a functional substitute for darkness in chronobiological experiments. While red light is generally considered less effective in eliciting circadian responses [29], studies have shown that, at sufficiently high intensities, it can acutely suppress melatonin synthesis in both rodents and humans [30,31]. The research mentioned above mainly concerns exposure to light of different wavelengths at night, while data on the effect of monochromatic light of different colors during photophase on pineal activity in mammals are largely limited.

Our previous studies demonstrated that monochromatic illumination during photophase significantly alters pineal indole metabolism in birds, with effects dependent on light color and intensity [32]. Pronounced changes occur in the course of the daily rhythm of MLT synthesis. Moreover, red light significantly increased synthesis of serotonin and its metabolites. In view of these findings, it seems reasonable to investigate the impact of light color during photophase on MLT synthesis-related indoles in mammals.

The aim of this study was to evaluate the effects of exposure to monochromatic light with varying wavelengths during the photophase on the metabolism of MLT-related indoles in the rat pineal gland. To investigate this, three groups of 3-month-old Wistar rats were exposed to 150 lx of blue, green, or red monochromatic light during the photophase for 7 days. A control group was maintained under white fluorescent light at the same intensity. Following the exposure period, the animals were euthanized at 3 h intervals over a 24 h cycle to collect data on their pineal indole metabolism.

## 2. Results

### 2.1. N-Acetylserotonin and Melatonin

**Figure 1:**
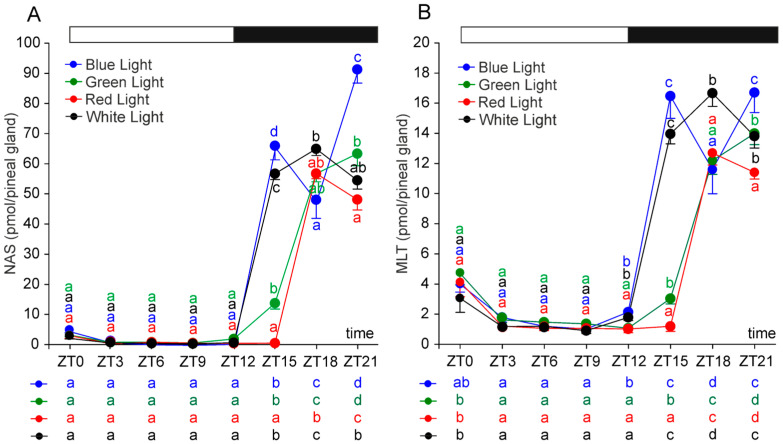
Contents (mean and SEM) of N-acetylserotonin (**A**) and melatonin (**B**) in the pineal glands of rats kept under monochromatic blue, green, and red light or fluorescent white light. The horizontal bar demonstrates the periods of light and dark phases of the daily cycle. The letters on the charts show statistically significant differences between groups at each time point. The letters below the charts show statistically significant differences between time points within a group. The same letters indicate means, which are not significantly different.

The diurnal pattern of pineal N-acetylserotonin (NAS) content differed significantly among rats exposed to monochromatic light of various wavelengths during the photophase, as well as in comparison to the control group (Figure 1A). At ZT 15 (three hours after the onset of scotophase), NAS levels in the red-light group remained at photophase values, whereas they were approximately 20-fold higher in the green-light group and over 100-fold higher in the blue-light and control groups compared with the photophase levels. At this time point, NAS content differed significantly between all groups, being the highest in the blue-light group and the lowest in the red-light group. By ZT 18, NAS levels in the red- and green-light groups had increased several-fold relative to ZT 15, reaching values similar to those observed in the control group.

In addition to differences in the timing of NAS elevation, the overall nocturnal pattern of NAS levels was strongly light-dependent (Figure 1A). In the control and red-light groups, the peak NAS level was observed at ZT 18, whereas in the green- and blue-light groups, the peak occurred at ZT 21. Notably, a significant decline in NAS was observed at ZT 18 in the blue-light group.

The daily patterns of MLT levels closely resembled those observed for NAS across all experimental groups, although the amplitude of changes was generally much lower (Figure 1B). During scotophase, MLT contents did not exceed photophase levels by more than 20-fold. The nocturnal increase in MLT levels was more pronounced in the control group and the blue-light group compared with the green- and red-light groups. Similar to NAS, the duration of elevated MLT levels was longer in the control and blue-light groups than in the green- and red-light groups. Peak MLT contents occurred at ZT 18 in the control and red-light groups and at ZT 21 in the green-light group. In the blue-light group, two distinct nocturnal peaks were observed—at ZT 15 and ZT 21.

### 2.2. Serotonin and Its Precursors

**Figure 2:**
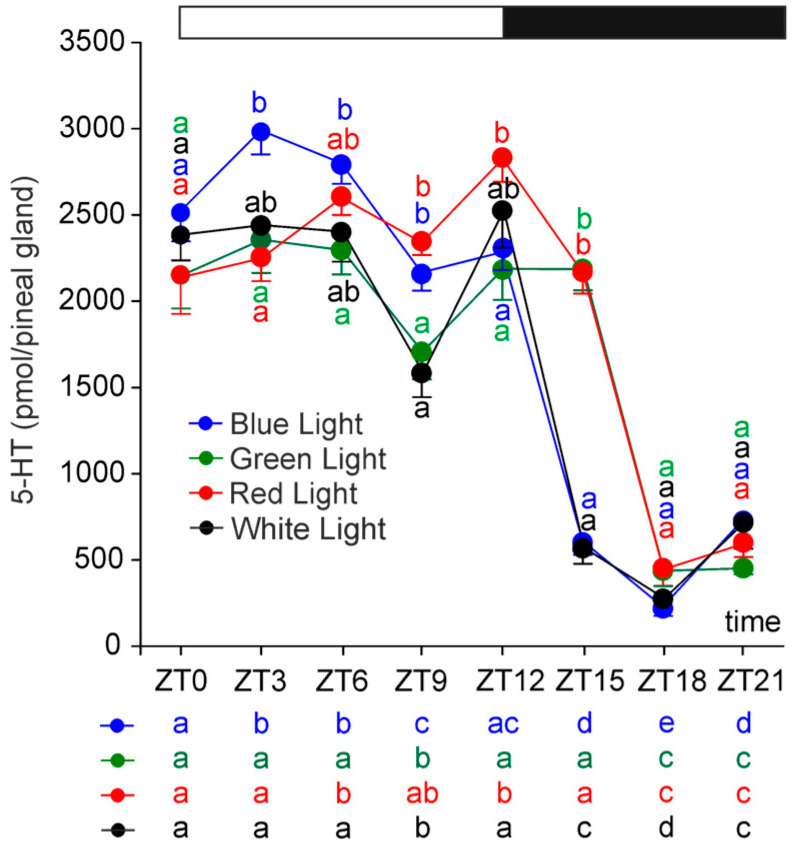
Contents (mean and SEM) of serotonin in the pineal glands of rats kept under monochromatic blue, green, and red light or fluorescent white light. The horizontal bar demonstrates the periods of light and dark phases of the daily cycle. The letters on the charts show statistically significant differences between groups at each time point. The letters below the charts show statistically significant differences between time points within a group. The same letters indicate means, which are not significantly different.

The content of serotonin (5-HT) exhibited marked diurnal fluctuations, characterized by an approximately fivefold decline during scotophase (Figure 2). The timing of these changes varied significantly depending on the photophase light conditions. In the control and blue-light groups, a reduction in 5-HT was first observed at ZT 15, whereas in the red- and green-light groups, it occurred later, at ZT 18. As a result, 5-HT levels at ZT 15 were significantly higher in the red- and green-light groups than in the control and blue-light groups. The 5-HT content returned to photophase levels at ZT 0 in all investigated groups of rats, so the duration of the nocturnal decline in the level of this amine was longer in rats exposed to white and blue light than in those exposed to green and red light. Notably, a reduction in 5-HT was also observed at ZT 9 across all groups. No significant between-group differences were found at ZT 18, ZT 21, or ZT 0; however, significant differences were detected during the photophase at ZT 3, ZT 6, ZT 9, and ZT 12.

**Figure 3:**
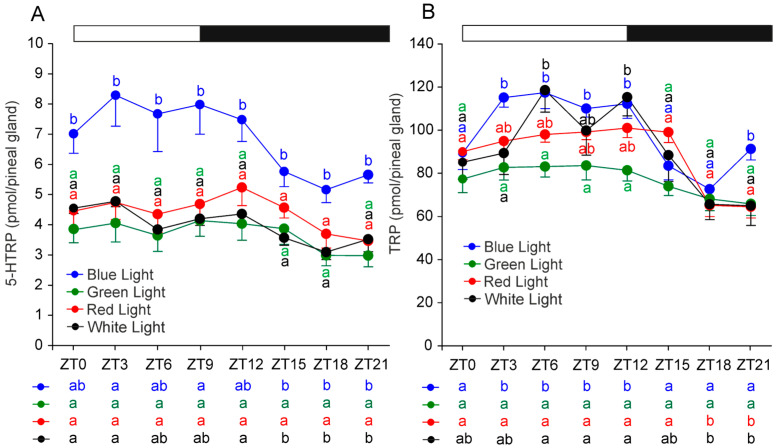
Contents (mean and SEM) of 5-hydroxytryptophan (**A**) and tryptophan (**B**) in the pineal glands of rats kept under monochromatic blue, green, and red light or fluorescent white light. The horizontal bar demonstrates the periods of light and dark phases of the daily cycle. The letters on the charts show statistically significant differences between groups at each time point. The letters below the charts show statistically significant differences between time points within a group. The same letters indicate means, which are not significantly different.

Significant daily variations in 5-hydroxytryptophan (5-HTRP), the direct precursor of 5-HT, were observed in the control and blue-light groups but were absent in the red- and green-light groups (Figure 3A). 5-HTRP levels at ZT 15, ZT 18, and ZT 21 were significantly lower than at ZT 0, ZT 3, and ZT 12 in the control group and than at ZT 3 and ZT 9 in the blue-light group. Across the day, 5-HTRP content was consistently higher in the blue-light group than in any other group.

Tryptophan (TRP) levels exhibited significant daily changes in all groups, except for the green-light group, where fluctuations were minimal (Figure 3B). In the control group, TRP levels during scotophase at ZT 18 and ZT 21 were significantly lower than during the photophase at ZT 6, ZT 9, and ZT 12. This nocturnal decrease was even more pronounced in the blue-light group, where TRP content was significantly lower at ZT 15, ZT 18, and ZT 21 compared with ZT 3, ZT 6, ZT 9, and ZT 12. In the red-light group, TRP was significantly lower at ZT 18 and ZT 21 compared with all other measured time points. During photophase, TRP levels in the green-light group were significantly lower than in the blue-light group (at ZT 3, ZT 6, ZT 9, and ZT 12) and the control group (at ZT 6 and ZT 12).

### 2.3. Metabolites of Serotonin Formed by Oxidative Deamination and Methylation

**Figure 4:**
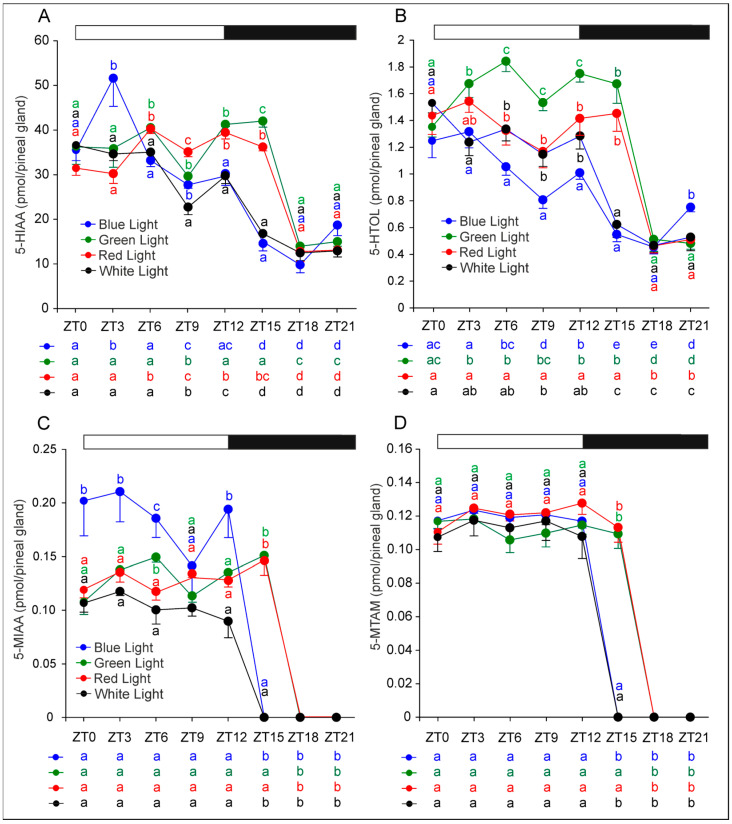
Contents (mean and SEM) of 5-hydroxyindoleacetic acid (**A**), 5-hydroxytryptophol (**B**), 5-methoxyindoleacetic acid (**C**), and 5-methoxytryptamine (**D**) in the pineal glands of rats kept under monochromatic blue, green, and red light or fluorescent white light. The horizontal bar demonstrates the periods of light and dark phases of the daily cycle. The letters on the charts show statistically significant differences between groups at each time point. The letters below the charts show statistically significant differences between time points within a group. The same letters indicate means, which are not significantly different.

The daily pattern of 5-hydroxyindoleacetic acid (5-HIAA) closely mirrored that of 5-HT (Figure 4A). In the control and blue-light groups, 5-HIAA levels were significantly lower at ZT 15, ZT 18, and ZT 21 compared with all other time points. In contrast, 5-HIAA levels at ZT 15 remained high in the red- and green-light groups, with significant reductions during scotophase observed only at ZT 18 and ZT 21.

Moreover, 5-HIAA levels were significantly higher at ZT 0, ZT 3, and ZT 6 than at ZT 9 and ZT 12 in the control group and at ZT 3 than at ZT 0, ZT 6, ZT 9, and ZT 12 in the blue-light group (Figure 4A). Conversely, in the red-light group, 5-HIAA levels were significantly higher at ZT 6, ZT 9, ZT 12, and ZT 15 than at ZT 0 and ZT 3. In the green-light group, photophase levels were relatively stable, with the exception of a significant decrease at ZT 9. Notably, at ZT 6, ZT 12, and ZT 15, 5-HIAA concentrations were significantly higher in the red- and green-light groups compared with the control and blue-light groups.

The daily fluctuations in 5-hydroxytryptophol (5-HTOL) were similar to those of 5-HIAA, but with pronounced between-group differences during photophase (Figure 4B). At ZT 3, 5-HTOL levels were significantly higher in the green-light group than in the blue-light and control groups. At ZT 6, ZT 9, and ZT 12, 5-HTOL levels remained significantly higher in the green-light group relative to all other groups. Additionally, at these time points, 5-HTOL levels were higher in the red-light and control groups compared with the blue-light group.

5-Methoxyindoleacetic acid (5-MIAA) was undetectable in the control and blue-light groups at ZT 15, ZT 18, and ZT 21, and in the red- and green-light groups at ZT 18 and ZT 21 (Figure 4C). At other time points, its levels remained very low. However, 5-MIAA content was significantly higher in the blue-light group than in other groups at ZT 0, ZT 3, ZT 6, and ZT 12.

5-Methoxytryptophol (5-MTOL) was undetectable at all time points in all groups.

Similarly to 5-MIAA, 5-methoxytryptamine (5-MTAM) was not detected at ZT 15, ZT 18, and ZT 21 in the control and blue-light groups, and at ZT 18 and ZT 21 in the red- and green-light groups (Figure 4D). Photophase levels of 5-MTAM were also consistently very low across all group.

## 3. Discussion

Research on rhythmic changes in pineal indole metabolism in mammals has primarily focused on MLT and two key enzymes responsible for its synthesis—arylalkylamine N-acetyltransferase (AA-NAT) and acetylserotonin O-methyltransferase (ASMT). In contrast, other indoles involved in MLT biosynthesis or those indirectly related to this pathway have received comparatively little attention, largely due to analytical limitations [33,34,35,36,37,38,39,40,41,42]. To the best of our knowledge, the present study is among the few that comprehensively examine all major MLT synthesis-related indoles within the same rat pineal gland [41,42].

Studies in birds have demonstrated substantial interspecies variability in the quantitative composition of pineal indoles, particularly in 5-HT and its downstream metabolites [43,44,45,46,47]. Notably, ducks and chickens exhibit fundamental differences in the mechanisms underlying the generation of diurnal rhythms of 5-MIAA and 5-MTOL, suggesting that these rhythms are not universally generated across species [43,44]. These findings underscore the importance of interpreting the diurnal changes and quantitative relationships among indole compounds observed in the present study within the broader context of data from other mammalian models.

Our results revealed significant daily fluctuations in the content of TRP and its derivatives, excluding 5-MTOL, which was undetectable. Comparison of the data obtained in rats kept under different light conditions revealed that these fluctuations were primarily driven by changes in the level of NAS synthesis. The nocturnal increase in NAS coincided with reductions in 5-HT, 5-HIAA, 5-HTOL, and 5-MIAA. The earlier onset of NAS synthesis at night matched the earlier decline in 5-HT and its metabolites. While TRP and 5-HTRP also decreased during scotophase, the changes were modest and less tightly correlated with NAS levels. The large 5-HT pool at the beginning of the night likely buffers the effects of increased NAS synthesis on TRP and 5-HTRP.

Similarly to our results, a nighttime decrease in TRP levels in the rat pineal gland was reported by Young and Anderson [42] and Frese et al. [38]. In contrast, TRP levels in Djungarian hamsters did not exhibit significant diurnal variation [39]. Data on tryptophan hydroxylase and 5-HTRP are limited and often inconsistent. Frese et al. [38] reported increased expression of tryptophan hydroxylase at night, while Deguchi [48] found no significant fluctuation in the enzyme activity across the day–night cycle. Higher 5-HTRP levels during the middle of scotophase compared with mid-photophase were reported in the rat pineal gland [38]. No rhythmic variation in 5-HTRP content was observed in Syrian hamsters under controlled lighting conditions [35]. In Djungarian hamsters, an increase in 5-HTRP during the first half of the night was found under a shorter photoperiod (14L:10D), but it was absent under a long photoperiod (16L:8D [34]. Significant day–night differences in 5-HTRP levels were not found in Djungarian hamsters maintained under natural photoperiods [39].

In contrast to its precursor, 5-HTRP, substantially more data are available regarding diurnal changes in 5-HT levels. In rats, pineal 5-HT content typically declines during the second half of the night [33,38]. A similar pattern has been observed in Syrian hamsters, where peak 5-HT levels occur during the latter part of the photophase and at the onset of scotophase [33,35,49]. In Djungarian hamsters, the daily rhythm of 5-HT exhibits photoperiod-dependent variability, indicating that environmental light conditions modulate its temporal profile [34]. The nocturnal decrease in 5-HT is generally attributed to its enhanced utilization by AA-NAT, whose enzymatic activity increases markedly during the night, facilitating the conversion of 5-HT into NAS [50]. Changes in 5-HIAA were generally parallel to those in 5-HT [33,40,49]. Although data on 5-HTOL are very limited, existing studies—consistent with our results—suggest similar rhythmicity to 5-HT and 5-HIAA [42].

A dramatic nocturnal rise in NAS levels in the rat pineal is well known [33] and likely among the highest observed in mammals. In contrast, MLT amplitude is lower, primarily due to the rate-limiting action of ASMT, which cannot convert all NAS into MLT. This enzymatic bottleneck has been observed in several species [43,44] but is particularly pronounced in rats [51,52]. The higher affinity of ASMT for NAS than for 5-HIAA and 5-HT likely contributes to the nighttime decline of 5-MIAA and 5-MTAM to undetectable levels. Published data on the pineal content of 5-MIAA and 5-MTAM in mammals are scarce [41,53,54,55]. The diurnal rhythm of 5-MTAM, similar to that of 5-HT, with higher levels during photophase, was described in the golden hamster [55]. Many more studies measured 5-MTOL in the mammalian pineal gland, however mostly using radioimmunoassay, and they reported the diurnal rhythm of 5-MTOL opposite to that of MLT [41,53,56,57].

**Figure 5:**
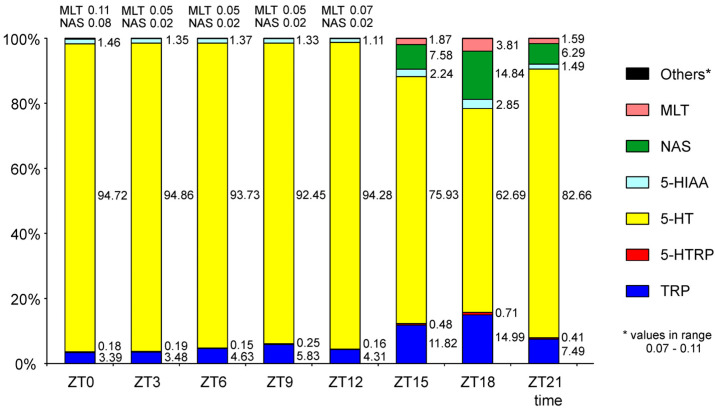
Percentage composition of indolic compounds in rat pineal glands collected at 3-h intervals over a 24 h cycle.

Analysis of the full panel of MLT-related indoles within a single pineal gland reveals several key observations. First, the rat pineal contains an exceptionally high proportion of 5-HT—exceeding 90% of total indoles during photophase and 60% during scotophase—providing a substantial reserve for nighttime NAS production (Figure 5). Second, 5-HT is minimally deaminated in rats, as indicated by high 5-HT:5-HIAA ratios (~50–60 in photophase, ~20–30 in scotophase). For comparison, ratios are much lower in other species: ~2 in Syrian hamsters [35], ~10–20 in Djungarian hamsters [34,39], and ~10 in European hamsters [40]. Third, ASMT activity is relatively low in rats, as is reflected in the ratio of NAS to MLT (approximately 4 in our study) and very low levels of 5-MIAA and 5-MTOL. Miguez and co-workers reported a NAS:MLT ratio of ~10 in rats [33] and ~0.2 or ~2 in Syrian hamsters [33,35]. In Djungarian hamsters, NAS levels were approximately twice those of MLT [34,39]. These interspecies differences underscore the need for further studies on MLT pathway heterogeneity, especially in non-rodent mammals.

Our data demonstrate, for the first time, that monochromatic light exposure during photophase significantly alters the nocturnal profiles of NAS and related indoles. In the green and red light groups, the nocturnal rise in NAS was delayed compared with the control and blue light groups. Furthermore, the increase in NAS was notably slower under red light than under green. These findings provide strong evidence for the critical role of blue light in the regulation of pineal indole metabolism. The blue spectral component present in the fluorescent lighting used in the control group was sufficient to elicit physiological responses comparable to those induced by monochromatic blue light, whereas the complete absence of blue light in the green and red light groups resulted in pronounced alterations in pineal indole metabolism.

The obtained results identify melanopsin-expressing retinal ganglion cells, which give rise to the retinohypothalamic tract, as the principal pathway mediating light-dependent regulation of indole metabolism. Melanopsin, a non-visual photopigment, is most sensitive to blue light, has moderate sensitivity to green light, and minimal sensitivity to red light [7,23]. The order of NAS and MLT increases observed during scotophase—fastest in the blue and control groups, intermediate in the green group, and slowest in the red group—corresponds well with this spectral sensitivity profile. However, visual photoreceptors may also contribute to mediating the effects of monochromatic light. As nocturnal animals, rats possess retinas dominated by rod photoreceptors containing rhodopsin, which is maximally sensitive to green–blue light, peaking at approximately 498 nm [58,59]. For color vision, rats have two types of cones: S-cones (short-wavelength, blue-sensitive) and L/M-cones (medium-to-long wavelength, red–green sensitive) [58]. Recently, Stritzel et al. [60] found that brief dim red light exposure at night produces strong activation of the suprachiasmatic nucleus master clock, rapid suppression of MLT secretion, and a subsequent phase shift in daily activity onsets in rodents. It should be noted that the complex network of synaptic connections within the retina may support multiple regulatory pathways capable of modulating circadian responses to different wavelengths of light [61]. Interpreting the effects of monochromatic light is further complicated by the physiology of suprachiasmatic nucleus neurons, whose responses to neurotransmitters depend on various factors, including the circadian phase [62,63,64,65]. This complexity and multifactorial nature of light-dependent circadian regulation suggest that both visual and non-visual photoreceptive systems may interact to shape the pineal indole rhythm.

The effect of monochromatic light on NAS synthesis is evidently a major factor driving the observed changes in the daily patterns of 5-HT and its metabolites. The absence of significant diurnal fluctuations in 5-HTRP levels in the red- and green-light groups can be attributed to reduced utilization of 5-HTRP for 5-HT synthesis, as the periods of elevated NAS production and decreased 5-HT levels were shorter in these groups compared with the blue-light and control groups. Nevertheless, some responses to monochromatic light—such as the consistently higher 5-HTRP levels observed in rats exposed to blue light—appear to involve additional, specific mechanisms that remain unclear.

Interestingly, pineal TRP levels during the photophase were significantly lower in the green-light group compared with both the blue-light and control groups. Although considerable attention has been given to the relationship between light intensity and seasonal affective disorder in humans, literature on the effect of light on TRP levels in the brain, including the pineal gland, or in blood is limited, providing little background for analyzing the mechanisms responsible for the phenomenon observed in the present study. Our results partially correlate with findings by Defrancesco et al. [66], who reported that the decrease in plasma TRP level induced by a TRP depletion procedure was significantly attenuated under bright light conditions (light temperature of 6298 K and intensity of 1530 lx) compared with dim light (temperature of 3493 K and intensity of 75 lx). However, other studies did not find a significant effect of light intensity on plasma TRP levels in women following TRP depletion [67]. Martyniuk et al. [32] reported no significant differences in plasma TRP levels in the pineal organs of domestic turkeys exposed to blue, red, and green monochromatic light during the photophase.

In our previous research, we characterized the expression of rhodopsin and melanopsin, as well as morphological alterations in the retinas of the same animals used in the current study [68]. We demonstrated that exposure to blue light significantly reduced the expression of both rhodopsin and melanopsin genes. Additionally, it induced morphological changes, including the loss or vesiculation of some photoreceptor outer segments and a reduction in the length of both outer and inner photoreceptor segments. Despite these retinal changes, the present results revealed no substantial differences in the profiles of daily fluctuations in NAS and MLT content between control rats and those exposed to blue light for seven days. The reduced expression of melanopsin appears to have remained sufficient to sustain photic signaling, thereby preserving normal circadian regulation under blue light exposure. Moreover, retinal photoreceptors seem to have the ability to discriminate between monochromatic blue light and full-spectrum light, as indicated by differences in 5-HTRP levels between rats in the blue-light and control groups. However, we cannot exclude the possibility that indole rhythms were more affected during the initial phase of blue light exposure (e.g., on the first day), potentially before the downregulation of melanopsin had taken effect. The observed downregulation of melanopsin expression in response to blue light likely represents a protective adaptation to sustained high-intensity exposure. A similar, though weaker, reduction in the expression of both photopigments was observed in rats exposed to green light. In contrast, red light exposure had no detectable effect on rhodopsin or melanopsin expression and caused no observable morphological changes in the retina.

## 4. Materials and Methods

### 4.1. Animals and Experimental Design

**Figure 6:**
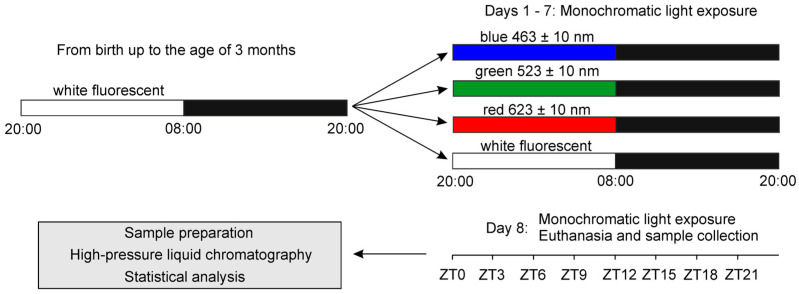
Schematic representation of the experimental design and animal management.

The female Wistar rats were housed in the chronobiological animal laboratory (Faculty of Veterinary Medicine, University of Warmia and Mazury in Olsztyn, Poland) under controlled temperature and humidity conditions compliant with animal welfare standards. They had free access to standard food and water. From birth up to 3 months of age, the rats were maintained in a 12/12 h light/dark cycle (light from 20:00 to 08:00) with white fluorescent illumination, approximately 100 lx at the level of the animal cages (Figure 6). After this initial period, the animals were randomly divided into four groups: three exposed to monochromatic blue (blue-light group), green (green-light group), and red (red-light group) light, and one control group (n = 32 rats per group). They were housed in transparent Plexiglas cages. The experimental groups were exposed to 12 h of their respective monochromatic light followed by 12 h of darkness for 7 days, while the control group continued in the light/dark cycle with white fluorescent light. Light periods ran from 20:00 to 08:00 and dark periods from 08:00 to 20:00. Monochromatic lights were emitted by LED strips—blue 463 ± 10 nm; green 523 ± 10 nm; red 623 ± 10 nm. White light in the control group was delivered via fluorescent lamps that emitted broad-spectrum illumination characterized by discrete spectral peaks. The light intensity at the animal head level was maintained at 150 lx, measured using either a Multi-Led (Tenmars, Taipei, Taiwan) light meter for monochromatic lights or a Standard ST-8820 Environmental Meter (Standard Instruments Co. LTD. Kwun Tong, Kowloon, Hong Kong) for white light. In addition, the power of LED light was determined using a laser power meter (Optik PT 9610, Gigahertz Optik GmbH, Munich, Germany), and similar values were obtained for all LED light exposures, i.e., 65–75 μW. The irradiance values varied between 3.3 and 3.8 W/m^2^ for red, green, and blue light.

For sample collection, four animals from each group were sacrificed in a CO_2_ chamber at eight specific time points throughout a 24 h cycle: 20:00 (ZT 0), 23:00 (ZT 3), 02:00 (ZT 6), 05:00 (ZT 9), 08:00 (ZT 12), 11:00 (ZT 15), 14:00 (ZT 18), and 17:00 (ZT 21). The pineal glands were frozen at −75 °C. During scotophase, samples were collected in dim red light (<5 lx) to minimize light exposure. All experimental procedures involving rats were conducted in full compliance with Polish and European Union regulations on animal welfare. The study protocol, including animal housing and euthanasia methods, was reviewed and approved by the Animal Welfare Committee of the Faculty of Veterinary Medicine, University of Warmia and Mazury in Olsztyn, Poland (Ethical approval for the project NCN 2017/01/X/NZ4/00838, dated 13 October 2017).

### 4.2. Indole Assay

#### 4.2.1. Chemicals

Standards: NAS, MLT, 5-HT, 5-HIAA, 5-MIAA, 5-MTOL, 5-HTRP, TRP, and 5-MTAM were purchased from Sigma-Aldrich (St. Louis, MO, USA). 5-HTOL was delivered by Santa Cruz Biotechnology (Dallas, TX, USA). Methanol of gradient-grade high-pressure liquid chromatography purity and perchloric acid were from Merck Millipore (Billerica, MA, USA). Sodium acetate, disodium EDTA, and acetic acid were obtained from J. T. Baker Chemicals (Center Valley, PA, USA).

#### 4.2.2. Sample Preparation

The pineal glands were sonicated in 60 μL of ice-cold 0.1 M perchloric acid using a Vibra-Cell VC 70 ultrasonic processor (Sonics & Materials Inc., Newtown, CT, USA) equipped with a 2 mm microtip probe to ensure complete tissue disruption. The homogenates were then incubated in an ice bath for 15 min to allow for protein precipitation, followed by centrifugation at 60,000× *g* for 15 min at 4 °C using an Allegra 64R centrifuge (Beckman Coulter, Indianapolis, IN, USA). The resulting supernatants were carefully collected and transferred into polypropylene autosampler vials (La-Pha-Pack Werner Reifferscheidt GmbH, Langerwehe, Germany) for subsequent chromatographic analysis. All samples were maintained in the autosampler at 5 °C and processed within 6 h of preparation to preserve analyte stability.

#### 4.2.3. High-Pressure Liquid Chromatography

The content of MLT synthesis-related indoles was measured by HPLC with gradient separation and fluorescence detection [43,46] using a Vanquish Duo U/HPLC system (Thermo Fisher Scientific, Waltham, MA, USA). The injection volume was 15 μL. The separation of indoles was performed using a Hypersil GOLD aQ column (150 × 4.6 mm, 3 μm particle size) (Thermo Scientific, Waltham, MA, USA) and a mobile phase, which was prepared by on-line mixing of methanol and an aqueous solution of 5 mM sodium acetate and 0.01 mM disodium EDTA (pH 4.5). The flow rate of the mobile phase was 1 mL/min. The initial methanol concentration was 3% (*v*/*v*), which was linearly increased to 30% (*v*/*v*) between 7 and 20 min of the separation and then kept constant. The recovery of methanol concentration to 3% occurred between 30 and 35 min of the separation, and the next sample was injected after 5 min. The fluorescence detection was performed with programmable changes in detector sensitivity at an excitation wavelength of 280 nm and an emission wavelength of 345 nm. The measurement of the 5-HTRP peak (the first peak) was performed at a detector sensitivity of 7, and thereafter the detector sensitivity was reduced to 1 (the minimum value) at 9 min of the separation to measure the 5-HT peak. Next, it was increased to 6 at 11.5 min of the separation to measure the TRP and 5-HIAA peaks and then to 8 (the maximum value) at 15.25 min to measure the peaks of other indoles. Chromeleon 7.2.10 software (Thermo Fisher Scientific, Waltham, MA, USA) was used for analysis of chromatograms. For all indoles, the limits of quantification (S/N ratio of 10:1 and RSD ≤ 15%) were below 2 pg per injection. The intra-day precision (RSD of peak area) was below 2%, and the inter-day precision was below 3%.

### 4.3. Statistical Analysis

Statistical analysis was performed using two-way analysis of variance (ANOVA), with light condition and sampling time as independent factors. Post hoc comparisons were conducted using Duncan’s multiple range test. Differences were considered statistically significant at *p* < 0.05. All analyses were carried out using Dell Statistica software (Version 13.1 PL; Dell Inc., Tulsa, OK, USA).

## 5. Conclusions

This study provides a comprehensive analysis of how monochromatic light exposure during the photophase influences the diurnal metabolism of MLT-related indoles in the rat pineal gland. The findings clearly demonstrate that light wavelength significantly affects the timing and progression of NAS and MLT rhythms. These changes were accompanied by concurrent alterations in 5-HT and its metabolites produced via oxidative deamination and methylation, indicating the strong upstream influence of NAS production on the broader indole metabolic pathway. Blue light most effectively preserved the typical rhythmicity observed under full-spectrum white light, whereas red light caused the most pronounced delay in nocturnal NAS and MLT synthesis.

These results highlight the pivotal role of melanopsin-expressing retinal ganglion cells in mediating wavelength-specific, light-dependent regulation of pineal activity. Nevertheless, the potential contribution of classical photoreceptors cannot be excluded. The study underscores the critical importance of light quality—particularly its spectral composition—in modulating circadian neuroendocrine function. These findings raise important questions regarding the physiological consequences of prolonged exposure to monochromatic lighting environments. Further studies are needed to explore species-specific responses, mechanistic pathways, and long-term outcomes of altered light spectra on circadian and pineal physiology.

## Data Availability

The raw data supporting the conclusions of this article will be made available by the corresponding author on request.

## Abbreviations

The following abbreviations are used in this manuscript:

5-HIAA	5-hydroxyindoleacetic acid
5-HT	serotonin
5-HTOL	5-hydroxytryptophol
5-HTRP	5-hydroxytryptophan
LED	light-emitting diode
5-MIAA	5-methoxyindoleacetic acid
5-MTAM	5-methoxytryptamine
5-MTOL	5-methoxytryptophol
AA-NAT	arylalkylamine N-acetyltransferase
ASMT	N-acetylserotonin O-methyltransferase
HPLC	high-pressure liquid chromatography
MLT	melatonin
NAS	N-acetylserotonin
TRP	tryptophan
